# 

**Published:** 1861-02

**Authors:** 


					﻿PublitUied Hontlily, at $3 per Anti tun in Advance.
z
fa
u
®
£
■
u
i*»
fa
fa
fa
57
i
fa
fa
i
a
fa
fa
cs
I z
e*
Z
fa
fa
&
i*
0
fa
s
fa
>»
i
fa
io
z'
fa
©
c
‘0
fa
fa
X
I
I
M
'*
X
I *
ATT.J A XT A.
.1 OU E NA L.
J. G. WESTMORELAND, M. D.,
Professor (u; Materia Medica and Medical Jcrisprvdence in the Atlanta Medical Coi.leg.,
EDITOR ANT) PROPRIETOR.
Pax et itcientia, wed verita* .ine llmorc.
Vol. VI. FEBRUARY, 1861.	No. VI.
A T I. A N T A, G A:
A.'XIL.A.T^TA INTELLIGENCES ILRIN’T.
i	1861.
____Bp	_	•
Each Xumber contain* Sixty-Four Page*.
"S
©
*
5
T
Z
T
»
S
»
1
£
«
£.
©
©
■S
X
JS
Q
X
•*
w*
n
z
0
$
E
U
o
s
1
5
z
I
»
*?
©
□
o
z
				

